# A Cure of Club Foot and One of Diseases of the Antrum

**Published:** 1847-03

**Authors:** 


*A Case of Club Foot and one of Diseases of the Antrum*, by Dr. Michel.—Dr. Myddleton Michel reported a case of congenital club foot, upon which he had operated with success. The patient, a little boy two years old, presented a kind of *talipes equinus* greatly complicated with *varus*, heel was much elevated, and the child walked on an artificial heel on the outer border of the foot, extending considerably upon its dorsal aspect.

From the mobility existing in the tibio-tarsal articulation, Dr. Michel entertained hopes of remedying the deformity without dividing the tibiales, the operation was therefore confined to the section of the tendo-achilles. This was performed by making a small *vertical* puncture with a lancet, one inch above the posterior extremity of the calcis, passing the blunt pointed tenotome on its flat side between the integuments and tendon, then dividing the tendon from behind forwards; after which the foot could, with a little effort, be almost brought into the normal position. Dr. M. called attention to this case, as signally exemplifying two points; 1st. the inutility in a great majority of instances, of dividing any other but the tendo-achilles, as the other structures then readily yield to a properly directed force. 2d. The importance of resorting to such an apparatus as should effect the same, without which auxiliary means, the cure is often missed. Martin's apparatus was used in this case.

Dr. M. Michel also brought before the notice of the Society, an interesting case of necrosis of the maxillary sinus. The subject is a child nine years old; the right cheek much swollen, face considerably disfigured, presenting a fistulous opening beneath the eye, through which a small probe may be made to pass into the antrum, and from thence into the mouth, through the alveolar processes which lodged the small molar and canine teeth; these having been removed in the early part of the disease.

After futile attempts to suppress the fetid discharge and to close the fistula, by the remedial agents usually employed, astringent injections, and pledgets of lint introduced into the antrum through the alveolar border, Dr. M. resorted to excision of that portion of the upper maxilla, extending from the medial line to the root of the molar process, removing all of the anterior wall of the antrum, and a great portion of the palatine process of the bone, with the vertical and rectangular cutting forceps, then, through the wide opening thus obtained, breaking down the spiculæ of dead bone contained within the sinus, and extracting several imperfectly developed teeth belonging to the second dentition, to the presence of which the disease, possibly, owed its origin. The patient doing well, redness and swelling of face dispersed, fistula beneath the eye healed, a crop of granulations springing up which will perhaps close the entrance into the sinus.—*South. Jour. Med. and Phar.*

